# Migrant Workers from the Eastern-Mediterranean Region and Occupational Injuries: A Retrospective Database-Based Analysis from North-Eastern Italy

**DOI:** 10.3390/ijerph16040673

**Published:** 2019-02-25

**Authors:** Matteo Riccò, Sergio Garbarino, Nicola Luigi Bragazzi

**Affiliations:** 1 Department of Prevention, Operative Unit for Health and Safety in the Workplaces—UOPSAL, Provincial Agency for Health Services of the Autonomous Province of Trento, 38123 Trento, Italy; mricco2000@gmail.com; 2 Department of Public Health, Occupational Health and Safety Unit—SPSAL, Azienda USL IRCCS di Reggio Emilia, 42122 Reggio Emilia, Italy; 3 Department of Neuroscience, Rehabilitation, Ophthalmology, Genetics, and Maternal/Child Sciences (DINOGMI), University of Genoa, 16132 Genoa, Italy; sgarbarino.neuro@gmail.com; 4 Postgraduate School of Public Health, Department of Health Sciences (DISSAL), University of Genoa, 16132 Genoa, Italy

**Keywords:** migrant worker, Ramadan fasting, circadian rhythm and biological clock, occupational injuries

## Abstract

The month of Ramadan is the ninth month of the Islamic lunar calendar, and, according to the Islamic tradition, it coincides with the month when the Noble Koran/Qur’an began to be revealed. In recent years, concerns about the potentially negative health effects of Ramadan fasting and the risks of work-related injuries have increased in Western European (EURO) countries. In the present study, we performed a retrospective database-based analysis assessing the impact of Ramadan fasting on occupational injuries (OIs) in North-Eastern Italy among migrant workers from the Eastern-Mediterranean Region (EMRO). Our results suggest that EMRO workers exhibit a significantly increased risk for OIs during Ramadan in periods characterized by heat-waves, while their frequency was somehow reduced for days associated with Ramadan characterized by increased but not extreme temperatures. However, these results may be attributable to an explanatory causation in the specific differences between EMRO and EURO workers in the job tasks performed at the workplace. Not coincidentally, no significant differences were found regarding industrial settings, mechanisms of OIs and final prognosis. Despite the obvious practical implications for health decision- and policy-makers, due to the limitations of the present investigation, further studies are warranted.

## 1. Background

Nowadays, around 10% of the Italian workforce is made up of migrant workers (about 2.3 million workers). Since the early 2000s, subjects from the member states of the World Health Organization (WHO) Regional Office for the Eastern Mediterranean (EMRO) have represented a relevant fraction of the migrant workforce, immediately after workers from Eastern Europe (particularly, Romanians and Albanians) [1,2]. Migrant workers are often associated with unskilled, highly physically-demanding jobs, and, as such, are affected by higher rates of occupational injuries (OIs) than their Italian counterparts [1,3].

Among the risk factors specifically associated with some migrant worker groups, and particularly with peoples from EMRO countries, concerns on the potentially negative health effects of Ramadan fasting and higher risks of work-related injuries have increased in highly developed Western European (EURO) Countries [4,5,6,7]. 

The month of Ramadan is the ninth month of the Islamic lunar calendar, and, according to the Islamic tradition, it coincides with the month when the Noble Koran/*Qur’an* began to be revealed. Observing Ramadan represents a religious duty (*as-sawm*), being one of the five pillars of Islam, and, historically, most of EMRO countries are characterized by high adherence to the traditional requirements of the religious fasting [8].

Since the Islamic calendar is lunar and not solar, the precise dates of the month of Ramadan change each year; furthermore, its beginning can vary slightly depending not only upon when the new moon is first sighted, but also based on regional customs and habits. As a consequence, Ramadan can last for 29 or 30 days, ending with a celebration feast termed as “*Eid al-Fitr*”. During this month, Muslim believers are requested to fast each day from sunrise to sunset. Also drinking liquids is strictly restricted. As such, fasting believers usually have one meal just before sunrise (named as *suhoor*) and an evening meal consumed immediately after sunset (*al-iftar*). In other words, Ramadan alternates moments of fasting with moments of re-feeding, being a time-restricted fasting or intermittent or circadian fasting [9,10,11]. In European settings, particularly in the Mediterranean area, Ramadan fasting may therefore coincide not only with the hottest weeks of the summer season, but also with a longer daytime [12,13]. Evidence suggests that the disruption of the internal biological clock due to the altered circadian rhythm, together with the fasting and the reduced water intake (potentially leading to dehydration), has the potential to severely impair the worker cognitively and/or physically, eventually increasing the risk of making mistakes, leading to accidents and injuries [14,15,16,17,18,19,20], especially among those subjects who would otherwise spend little time outdoors, such as part-time or seasonal workers [14,15,16,17,18,19,20,21].

Focusing on Western European countries, and particularly on Italy-based workers from EMRO countries, it should be stressed that fasting and circadian rhythm disruption have an impact on a subset of the workforce that, because of the high prevalence of unskilled, dangerous, and demanding jobs [1], has the potential to be particularly affected by climate changes and the global rise of environmental temperatures [14,15,16,17,18,19,20,21,22,23]. Interestingly enough, such concerns are also ever more felt and perceived not only in EURO countries, where adaptations of the working shifts and rhythms to the timing of the Ramadan fasting are implemented by entrepreneurs and enterprises without the support of institutions and governmental bodies [4,6,9,10,11,24,25]. For instance, some religious authorities, such as the Egyptian theologian Yusuf al-Qaradawi, chairman of the “International Union for Muslim Scholars” (IUMS) and “Al Qaradawi Centre For Islamic Moderation and Renewal” based in Doha (Qatar), have recently suggested that religiously fasting workers should be “permitted to break their fast, especially if they feel very, very thirsty”.

Interestingly enough, from a scientific standpoint, despite the high international concern, very little is currently known about the occupational impact of fasting during the month of Ramadan. The aim of this study is therefore to investigate the OI risk differentials between EMRO migrant workers and EURO natives, taking into account individual (age) and job characteristics, as well as meteorological data and the possible influence of Ramadan fasting in a North-Eastern Italian region extensively involved in climate change in the last decade.

## 2. Material and Methods

### 2.1. Setting

Trentino, officially designated as the “Autonomous Province of Trento” (APT), together with South Tyrol, is one of the two provinces that make up the region of Trentino-Alto Adige/*Südtirol*. More in detail, APT is located in Italy’s North East, and covers a total area of 6214 km^2^ (2399 mi^2^), with a total population of 537,416 inhabitants, according to the 2015 Census statistics.

Located in the Mediterranean sub-alpine region of Northeastern Italy, APT has recently experienced a 0.9 °C increase in average temperatures from 1971–2000. Summer temperatures have been particularly affected, as the year incidence of “warm days”, “warm nights” (that is to say, days and nights having average temperatures above the 90th percentile of the reference period), and “tropical nights” (i.e., nights with minimum air temperature greater than 20 °C) have nearly doubled since 1970–2000, raising concerns concerning heat exposure in the workplace. 

According to the available data and labor force statistics from the Statistical Institute of the Autonomous Province of Trento (ISPAT), in the last decade, the workforce encompassed around 250,000 adult aged subjects per year. Approximately one fifth of them (46,454) are foreign-born and, according to the Annual Report from the APT, up to 20% of all migrants are from countries of the EMRO region (i.e., 3.7% of total workforce) [14]. 

### 2.2. Occupational Injuries

Data about OIs for the APT from 2000 to 2013 were retrieved from the institutional archive of the Operative Unit for Health and Safety in the Workplaces (UOPSAL: *Unità Operativa di Prevenzione e Sicurezza negli Ambienti di Lavoro* in Italian), the governmental service representing the local structure for the management and prevention of OIs, occupational diseases, and work-related diseases in the workplaces. The information flow includes data from local emergency departments, local safety authorities (e.g., municipalities, Police, etc.) and information from compulsory Occupational Insurance (e.g., National Insurance for Occupational Illness and Injuries, INAIL). Occupational insurance in Italy is compulsory for all activities that the law defines as risky; it protects workers against damages due to work-related accidents, OIs and occupational diseases.

In case of duplicate data (i.e., the same event was reported by several sources), only the last record was included in the analysis. While only events causing more than 3 days’ absence from work are usually compensated and recorded by Occupational insurance data, integration of informatory flow has made data available regarding any accident occurring at work and causing a trauma to one or more people, irrespective of its prognosis.

Available data were anonymized in order to include only age at the time of the event, sex, and country of birth, and incorporated reference to the geographical site (municipality-level detail) and calendar date of the events, the nature of OIs, bodily location, mechanism, and agency of injury/disease [14].

For further details, the reader is referred to [21].

### 2.3. Metereological Data

Meteorological data, including daily average (T_day_), minimum (T_min_), maximum (T_max_) temperatures, air relative humidity, atmospheric pressure, wind speed and solar irradiation for the study period, were obtained from the Meteotrentino Service of the APT. The Meteotrentino Archive includes data from a total of 214 meteorological stations scattered over the provincial area, allowing us to directly link the geographical site of OIs with air temperature at the time of the accident. As data about air relative humidity, wind speed and solar irradiation were not available for all meteorological stations, data from the nearest station at the time of the index OIs were ultimately retrieved. 

Calendar days were initially categorized in a warm (April to September) vs. cold season (October to March), then by T_min_ and T_max_ as follows: namely, frost days (i.e., days with T_min_ < 0 °C), summer days (i.e., days with T_max_ > 25 °C), and summer days/tropical nights (i.e., days with T_max_ > 25 °C and T_min_ > 20 °C). Days not included in the aforementioned definition were classified as “neutral days”. In order to assess to potential effect of heat-waves (HW) on OIs, the whole observation period was then dichotomized as HW time period vs. non-HW time period. Currently, there is no universal definition of HW, although it may be broadly defined as a prolonged period of excessive heat [26]. In order to more easily compare our results with similar types of research, we have defined a HW event as a time period including at least 3 consecutive days with T_max_ ≥ 35 °C.

### 2.4. Statistical Analysis

Before commencing any statistical processing and handling of data, figures were visually inspected for potential outliers. Categorical variables were computed as percentages, where appropriate. Association of reported OIs during the Ramadan-time period with being a migrant worker from EMRO country vs. being a EURO worker was initially assessed through chi-squared test.

Comparisons were performed by occupational settings (i.e., manufacturing; finance, property and business services; wholesome and retail trade; transport, storage and post; agriculture; services, including health services; and construction industry, that was assumed as the reference category), by hour of the day (categorized as: 01.00–06.00; 13.00–18.00; 19.00–24.00, and 7.00–12.00 that, as suggested by Garbarino et al. [27] was assumed as the reference category), by meteorological characteristics of the calendar day (categorized in: frost day; summer day; tropical night; and neutral day that was assumed as the reference category), occurrence vs. absence of HW, and kind of OIs.

In this regard, as we analyzed the occupational impact of Ramadan with particular attention to the potential risks at the workplace, we focused on events more frequently associated with inattention and impairment of balance/gait (i.e., falls to lower level; falls to same level; injury occurred during manual handling; and injury occurred during use of tools/machineries, that was defined as the reference category), rather than on the clinical characteristics or anatomical sites.

Also, prognosis was included in the analysis and dichotomized as <40 days vs. ≥40 days. Such categorization was implemented following the framework of the Italian law, as OIs having a prognosis greater than 40 days may imply automatic prosecution by local Prosecutor’s office in order to identify any criminal responsibility. Eventually, Ramadan fasting was dichotomized as occurring during North-Italian warm season (mid June–mid September) vs. North-Italian cold season (mid September–mid June). A multivariate logistic regression analysis was then carried out by adjusting the model for the sex and age of the worker, with calculation of Odds Ratios (OR) and their respective 95% Confidence Intervals (95% CI).

In the analyses including meteorological data (i.e., daily temperatures), data on air relative humidity, atmospheric pressure, wind speed and solar irradiation were included as covariates.

All statistical analyses were carried out with the commercial software “Statistical Package for the Social Sciences” (SPSS for Windows version 25, IBM Corp, Armonk, NY, USA). 

Figures with *p*-value less than 0.05 were considered statistically significant. 

### 2.5. Ethical Approval

The study included only a retrospective assessment of data available through an Institutional Database, and the analyses on OI rates were performed as a part of the periodic epidemiological survey on occupational health and safety risks. 

Personal data was restricted to information about the OIs, and was treated in order to guarantee the respect of privacy of the involved workers, as specifically stated by Italian Law No. 675 of 1996 on personal data protection. Therefore, the study did not require any preliminary evaluation by the local Ethical Committee.

## 3. Results

Numbers of workers’ injury claims in the APT between 2000 and 2013 are reported in Table 1, and are broken down according gender, age group, WHO region of origin, industrial settings, kind of OIs and prognosis. 

More in detail, a total of 147,024 OIs occurred during the study period, 13,150 (8.9% of all OIs) during the Islamic Holiday (IH) time-period (8646 during the cold seasons and 4504 during the warm seasons, 65.7% and 34.3% respectively).

As shown in Table 2, stratifying according to the WHO region of origin, 135,982 OIs affected workers from the EURO region (92.5% of all OIs, 12,061—91.7%—during the IH period and 7948—91.9%—and 4113—91.3%—during the cold and warm seasons, respectively), whereas 6203 OIs involved affected workers from the EMRO region (4.2% of all OIs, 617—4.7%—during the IH period and 407—4.7%—and 210—4.7%—during the cold and warm seasons, respectively).

In the univariate analysis (Table 3), during the IH period, workers from the EMRO region had more frequent claims for OIs than workers from the EURO region (*p* = 0.004; OR 1.135 [95% CI 1.042–1.236]). Stratifying according the season, OR was 1.133 [95%CI 0.984–1.340] during the warm season (*p* = 0.084, borderline significant) and 1.136 [95% CI 1.025–1.259] during the cold season (*p* = 0.015). In the multivariate analysis, correcting for gender and age group, the adjusted OR (adjOR) resulted 1.131 ([95% CI 1.038–1.231], *p* = 0.005) for OIs occurring during IH, while adjORs stratified according to cold and warm seasons were 1.114 ([95% CI 1.005–1.235], *p* = 0.041) and 1.182 ([95% CI 1.026–1.362], *p* = 0.021), respectively. 

In the multivariate logistic regression analysis (reported in Table 4), no significant difference was identified regarding the occupational settings. For instance, also more physically demanding occupational settings such as in the manufacturing industry (adjOR 0.781 [95% CI 0.603–1.010], *p* = 0.603) and agriculture (adjOR 0.638 [95% CI 0.269–1.510], *p* = 0.307) were associated with non-statistically significant increased risk for the IH season. Focusing on the time of the OIs, assuming the time period of 07.00–12.00 as the reference category, the statistical analysis showed that during the IH period, 13.00 to 18.00 was associated with a significant increased of OIs (adjOR 1.385 ([95% CI 1.112–1.726], *p* = 0.004).

**Figure 1:**
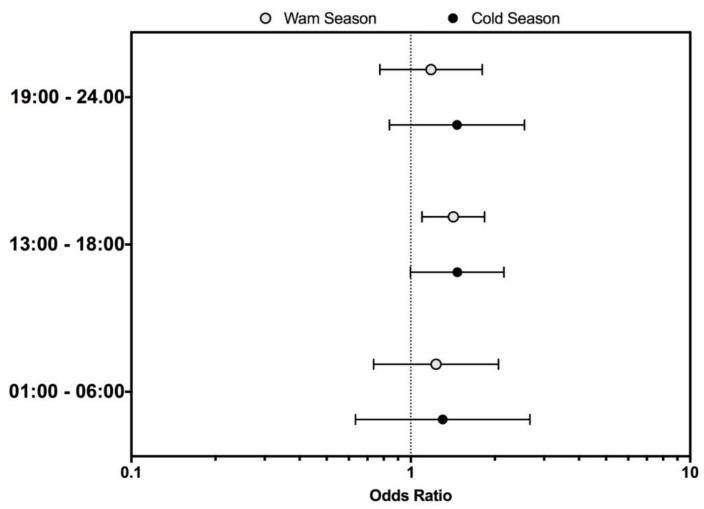
Occupational injuries (OIs) among the EMRO workers stratified by time of day and the season of occurrence in the Autonomous Province of Trento (APT) between 2000 and 2013.

**Figure 2:**
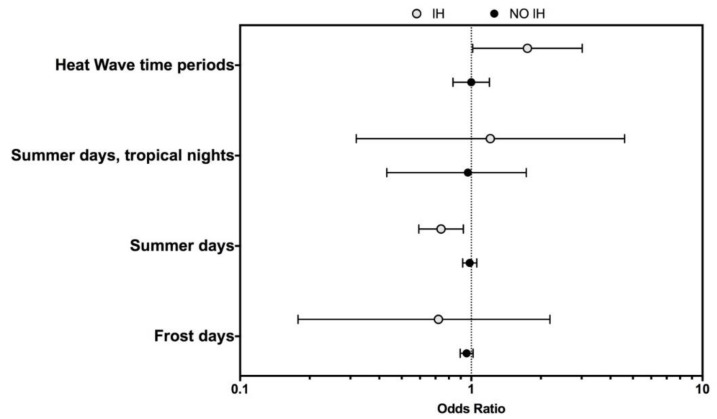
Occupational injuries (OIs) among the EMRO workers stratified by meteorological characteristics of the calendar day of occurrence in the Autonomous Province of Trento (APT) between 2000 and 2013 broken down by Islamic Holiday time-period (IH) vs. not IH.

Interestingly enough, when we stratified by the season of occurrence, the increased risk was limited to the IH period occurring during the warm season (i.e., adjOR 1.420 [95% CI 1.097–1.838]), as shown in Figure 1. In contrast, when data were stratified by the meteorological data of the index day (Figure 2), while no significant differences were found for no regular days, we found a reduced occurrence of OIs in EMRO workers on days fulfilling the definition of ‘summer days’ occurring during the IH period (i.e., adjOR 0.740 [95% CI 0.593–0.924]). Summer days with tropical nights during the IH time-period had a not significantly increased occurrence of OIs in EMRO workers (i.e., adjOR 1.209 [95% CI 0.318–4.602]), while the increase was significant for days associated with the HW time-period during IH (i.e., adjOR 1.749 [95% CI 1.014–3.017]).

Meteorological variables (namely, maximum daily temperature, minimum daily temperature, average daily temperature, relative humidity, solar radiation, atmospheric pressure and wind speed) are shown in Table 5.

Stratifying according to WHO region and meteorological exposures, EMRO workers were exposed to more extreme conditions than their EURO counterparts (*p* = 0.003 for atmospheric pressure, *p* = 0.047 for wind speed, *p* = 0.020 for solar radiation). Further details are shown in Table 6.

## 4. Discussion

Our study identified a significantly increased risk for OIs in EMRO workers during IH occurring in HW time period, while their frequency was reduced for days associated with IH characterized by increased but not extreme temperatures. These results are somehow consistent with previous reports from the same geographical regions, that suggested that an increased risk of agricultural OIs is associated with higher environmental temperatures [21,26,28]. Moreover, we identified a possible “risk window” in the timeframe 13:00–18:00, but only during the “warm season”, when the impact of altered feeding and sleeping habits is supposedly greater because of the late or even very late sunset time, and water deprivation which overlaps with higher environmental temperatures. In contrast, some or all of our results (i.e., the somehow reduced risk for OIs during warm, not extreme temperatures) are apparently in contrast with the basic assumption of a potentially detrimental effect of fasting and disruption of circadian rhythms on worker safety that is otherwise suggested by the increased risks for OIs during HW time periods. However, such results may be explained differently. It is reasonable that workers requested to perform their duties in an unfavorable climate may delay more risky habits and daily tasks when possible [20,21]. As a consequence, the reduced risk we identified compared with the HW time period may be a sort of inverted harvesting phenomenon, with workers restrain from certain daily tasks until the specific characteristics of their job eventually force them to work in uncomfortable temperatures, with subsequent increased risk for OIs [20,21,28,29]. It is well known that Ramadan fasting can disrupt circadian rhythms and, as such, can profoundly impact on sleeping patterns, thus negatively influencing wakefulness at the working place. A recent study by Ajabnoor and colleagues [24] has shown that Ramadan fasting impairs Circadian Locomotor Output Cycles Kaput (CLOCK) expression. 

However, a critical review carried out by Qasrawi et al. [22] has found discrepancies and contrasting findings in the existing scholarly literature, as studies that controlled for environmental factors and sleep/wake schedules reported no significant disturbances in sleep patterns, dynamics and architecture. Nevertheless, several studies have consistently reported that the main change in sleep architecture during fasting is a reduction in the proportion of rapid eye movement (REM) sleep. Despite the mixed findings regarding the impact of Ramadan fasting on sleep, from a clinical standpoint, many studies suggest decreased daytime alertness during Ramadan in Islamic countries. 

Khalfallah and collaborators [7] compared the physical workload during and outside the period of Ramadan by a continuous recording of the heart rate during work. The authors found that the physical workload measured as relative cardiac cost was higher during the month of Ramadan. However, such data require a critical analysis. On the one hand, it should be stressed that in Islamic countries, daytime activities and working hours are often adapted to the changed rhythm during IH. For instance, in the very strictly Islamic Saudi Arabia, during Ramadan, a Muslim cannot work more than six hours a day, and Saudi labor laws require work stoppage at construction sites when the temperature reaches 50 °C.

On the other hand, it is not unusual for workers contending with high heat and humidity to ultimately break their IH fasting to avoid health problems, and such an approach has been recently supported by official statements from religious authorities in the United Arab Emirates. In Western countries, as well as in some secularized countries with a significant Islamic background, work hours remain substantially unchanged; adapting working schedules to IH has the potential to elicit conflicting interactions with workers from other cultural backgrounds [30].

Some European employers have expressed concerns about the alertness of Muslim employees while performing potentially dangerous activities or maintaining concentration during mentally straining tasks. However, literature on the topic is scarce, in that very few studies have addressed it. Among these studies, in the Federal Republic of Germany (FRG) Schmahl and coworkers [4] were able to observe moderate-to-severe health disturbances in Muslim (especially Turkish) workers performing heat work and other heavy labor during the IH. More recently, Mertens and collaborators [5] in Belgium found that, in a sample of 20 fasting workers, daytime sleepiness moderately increased, whereas neuro-performance remained unaffected and concentration improved with large inter-individual differences. Other studies have investigated this topic in Arabic countries, reporting similar findings [6,8,25,31,32].

Our study suffers from a number of limitations that should be properly recognized. First at all, data regarding the OIs were potentially affected by some inaccuracies. Available information about occupational injuries was retrieved from several sources, whose reliability may be intrinsically questioned because of reporting bias and the heterogenous quality of the reported data. Second, it should be stressed that while the prognoses included in the data from emergency departments and local municipalities is usually reliable, institutional information flow usually includes the definitive information. However, we included only consolidated estimates, when available. Third, as original data sources were not specifically designed for the analysis of occupational risk factors, only rarely did it included some hints about environmental and individual risk factors for OIs (e.g., the level of physical activity performed at the time of the event, the personal protective equipment possibly worn at time of the OIs, hydration status, drug use assumed before the event, etc.) [21,28]. Eventually, as our analysis included only institutional data, it is reasonable to assume that minor events, whose actual prognosis does not require any sick leave, particularly those occurring among self-employed workers, may not have been recorded, potentially leading to underestimations of actual risk compared with more severe OIs.

Another major drawback was due to the fact that we used the WHO EMRO region of origin as a proxy of being Muslim and performing religious fasting. Information flows usually include the country of origin of the injured worker, enabling only schematic analysis. Unfortunately, available data on workforces are not fully comparable, as more common denominators on OI studies (e.g., annual number of workers or full time equivalents or person time at-risk) are not usually categorized by country of origin, usually including the “nationality” of the injured worker (and usually as “Italian” vs. “Not-Italian”). As a foreigner residing and working in Italy for at least 10 years may obtain Italian nationality; including such a factor in the analyses would have added even more uncertainty to the final data. As a consequence, our results presented a comparison of the frequency of events; such comparisons between EMRO workers and EURO workers should be interpreted with caution because of the heterogenous composition and numerosity of these two groups of workers.

Focusing on the religious factor by itself, on the one hand, it is undeniable that religiosity is, indeed, a complex, multi-factorial construct which does not necessarily coincide with geographic origin. Moreover, as religious identity is not customarily recorded in clinical data, and some countries in the EURO region have a significant Muslim population (e.g., Albania, Bosnia Herzegovina, Kosovo), the identification of both a reference population and an at risk group may have been affected by a significant bias. On the other hand, it is true that the fasting during the month of Ramadan is, the most observed among the five pillars of Islam, also by “lay” or little practicing Muslims, similarly to the religious holidays of other religions (e.g., for Western countries, Christmas and Easter). Eventually, it should be kept in mind that the main stressors associated with the increased risk for OIs during alternate fasting are fluid depletion, physical and/or cognitive impairment, and environmental heat [14,15,16,17,18,19,20,21,28].

In our study, we assessed environmental factors by dichotomizing the timeframe in IH occurring in the warm vs. the cold season, and analyzed the environmental stressors through meteorological data. Unfortunately, we were unable to recall the actual exposure to environmental stressors of assessed workers. In other words, since in our case the definition of migrant workers and potential fasteners during the IH time-period substantially overlapped, and migrant workers are usually associated with more strenuous, physical demanding occupational settings both in agriculture and industries, it is possible that the increased risk for OIs we found in the timeframe 13.00–18.00, as well as the increased risk for OIs during HW, may be concurring remarks rather than a direct causation. In other words, it is reasonable that we identified an increased risk for EMRO workers to perform strenuous tasks in uncomfortable conditions rather than an increased risk of OIs occurring because of fasting and altered sleeping habits per se [1,21].

## 5. Conclusions

In a globalized society, more and more Muslim workers are working in Western countries. Our results suggest that during Ramadan, EMRO workers exhibit a small but significant increased risk for OIs during the hottest hours of the summer days, particularly during HW. However, this difference may be due to an explanatory causation in the specific differences between EMRO and EURO workers in the job tasks performed at the workplace. Not coincidentally, no significant differences were found regarding industrial settings, mechanisms of the injury and final prognosis. Despite the obvious practical implications for health decision- and policy-makers, due to the above-mentioned limitations, further studies are warranted.

## Figures and Tables

**Table 1:** Numbers of occupational injuries (OIs) that occurred in the Autonomous Province of Trento (APT) between 2000 and 2013.

Classification	All Retrieved OIs	
	Number	Percentage
**Total**	147,024	100.0%
**Gender**		
Male	114,470	77.8%
Female	32,557	22.1%
**WHO Region of Origin**		
EURO	135,982	92.5%
EMRO	6203	4.2%
PAHO	2653	1.8%
SEARO	352	0.2%
WPRO	332	0.2%
AFRO	1502	1.0%
**Age Group**		
≤24	26,619	18.1%
25–34	36,330	24.7%
35–44	40,114	27.3%
45–54	30,802	20.9%
≥55	13,131	9.0%
**Industrial settings**		
Manufacturing	27,524	18.7%
Finance, Property and Business Services	25,576	17.4%
Wholesale and Retail Trade	6099	4.1%
Transport. Storage and Post	5223	3.6%
Agriculture	6726	4.6%
Construction	22,346	15.2%
Services, including Health Services	5196	3.5%
Other and undetermined	48,256	32.8%
**Kind of injury**		
Falls to a lower level	8543	5.8%
Fall to same level	23,448	15.9%
Manual handling	20,050	13.6%
Use of tools/machinery	28,558	19.4%
Other	66,457	45.2%
**Prognosis**		
<40 days	128,627	87.5%
≥40 days	18,299	12.4%
Death	130	0.1%

Abbreviations: AFRO (Africa); EMRO (Eastern Mediterranean); EURO (Europe); OI (occupational injury); PAHO (Pan American Health Organization); SEARO (South East Asia); WHO (World Health Organization); WPRO (Western Pacific).

**Table 2:** Numbers of occupational injuries (OIs) that occurred in the Autonomous Province of Trento (APT) between 2000 and 2013, by WHO region of origin (EURO vs. EMRO) and by time period, comparing injuries occurring in the Islamic holiday time-period (IH), as a whole and dichotomized in cold season IH (mid September–mid June), and warm season IH (mid June–mid September).

	EURO (REF)			EMRO		
	TOTAL	IH Cold Season	IH Warm Season	TOTAL	IH Cold Season	IH Warm Season
	No. (%)	No. (%)	No. (%)	No. (%)	No. (%)	No. (%)
**Total**	135,982 (100.0%)	7948 (100.0%)	4113 (100.0%)	6203 (100.0%)	407 (100.0%)	210 (100.0%)
**Gender**						
Male	105,218 (77.4%)	6309 (79.4%)	3085 (75.0%)	5622 (90.6%)	374 (91.9%)	191 (91.0%)
Female	30,735 (22.6%)	1637 (20.6%)	1028 (25.0%)	581 (9.4%)	33 (8.1%)	19 (9.0%)
**Age Group**						
≤24	25,221 (18.5%)	1616 (20.3%)	619 (15.0%)	768 (12.4%)	52 (12.8%)	24 (11.4%)
25–34	32,676 (24.0%)	2054 (25.8%)	870 (21.2%)	2128 (34.3%)	155 (38.1%)	61 (29.8%)
35–44	36,170 (26.6%)	2134 (26.8%)	1119 (27.2%)	2267 (36.5%)	148 (36.0%)	65 (31.0%)
45–54	29,008 (21.3%)	1468 (18.5%)	1012 (24.6%)	935 (15.1%)	50 (12.3%)	53 (25.2%)
≥55	12,907 (9.5%)	676 (8.5%)	493 (12.0%)	105 (1.7%)	2 (0.5%)	7 (3.3%)
**Industrial settings**						
Manufacturing	24317, 17.9%	1438, 18.1%	611, 14.9%	1928, 31.1%	114, 28.1%	55, 26.2%
Finance, property and business services	23,524 (17.3%)	229 (3.2%)	118 (2.9%)	1038 (16.7%)	26 (17.0%)	44 (21.0%)
Wholesame and retail trade	5746 (4.2%)	311 (3.9%)	171 (4.2%)	165 (2.7%)	18 (4.4%)	2 (1.0%)
Transport, storage and post	4743 (3.5%)	258 (3.2%)	118 (2.9%)	268 (4.3%)	13 (3.2%)	9 (4.3%)
Agriculture	6554 (4.8%)	63 (0.8%)	597 (14.5%)	80 (1.3%)	0 (-)	6 (2.9%)
Construction	20,947 (15.4%)	1,326 (16.7%)	628 (15.3%)	946 (15.3%)	72, 17.7%	32 (15.2%)
Services, including health services	4969 (3.7%)	229 (2.9%)	223 (5.4%)	34 (0.5%)	0, -	1 (0.5%)
Other and undetermined	45,075 (33.2%)	3016 (37.9%)	983 (23.9%)	1742 (28.1%)	120 (29.9%)	61 (29.0%)
**Kind of injury**						
Falls to a lower level	7906 (5.8%)	610 (7.7%)	101 (2.5%)	355 (5.7%)	30 (7.7%)	3 (1.4%)
Fall to same level	21,914 (16.1%)	1406 (17.7%)	599 (14.6%)	844 (13.6%)	65 (16.0%)	24 (11.4%)
Use of tools/machineries	23,648 (19.1%)	2063 (26.0%)	495 (12.0%)	1330 (21.4%)	118 (29.0%)	22 (10.5%)
Manual handling	16,612 (13.4%)	1055 (13.3%)	680 (16.5%)	961 (15.5%)	66, 16.2%	24 (11.4%)
Others	56,557 (45.6%)	2814 (35.4%)	2238 (54.4%)	2713 (43.7%)	128 (31.4%)	137 (65.2%)
**Prognosis**						
<40 days	118,723 (87.3%)	7002 (88.1%)	3524 (85.7%)	5510 (88.8%)	365 (89.7%)	180 (85.7%)
≥40 days	17,136 (12.6%)	936 (11.8%)	586 (14.2%)	690 (11.1%)	42 (10.3%)	30 (14.3%)
Deaths	130 (0.1%)	10 (0.1%)	3 (0.1%)	3 (0.0%)	0 (-)	0 (-)

Abbreviations: EMRO (Eastern Mediterranean); EURO (Europe); IH (Islamic Holiday).

**Table 3:** Comparison of prevalence rates for OIs in EMRO vs. EURO workers by time-period, i.e., occurring in the Islamic holiday time-period (IH), as a whole and dichotomized in cold season IH (mid September– mid June), and warm season IH (mid June–mid September).

	EMRO	EURO (REF)	Statistical Significance (*p*-Value)	OR (95%CI)	adjOR (95%CI)
IH (total)	617, 9.9%	12,061, 8.9%	0.004	1.135 (1.042–1.236)	1.131 (1.038–1.231)
IH cold season	407, 6.6%	7948, 5.8%	0.015	1.136 (1.025–1.259)	1.114 (1.005–1.235)
IH warm season	210, 3.4%	4113, 3.0%	0.084	1.133 (0.984–1.340)	1.182 (1.026–1.362)

Abbreviations: adjOR (multivariate analysis adjusted odds-ratio); CI (confidence interval); EMRO (Eastern Mediterranean); EURO (Europe); IH (Islamic holiday); OR (odds-ratio); REF (reference).

**Table 4:** Comparison of prevalence rates for OIs in EMRO workers by settings of the injury and time period, i.e., occurring in the Islamic holiday time-period (IH) vs. non IH.

	IH SeasonNo. (%)	Rest of Calendar Year No. (%)	*p*-Value	adjOR	95%CI	
Industrial settings						
Manufacturing	169 (27.4%)	1759 (31.5%)	0.603	0.781	0.603	1.010
Finance, property and business services	113 (18.6%)	925(16.6%)	0.725	1.055	0.784	1.419
Wholesale and retail trade	20 (3.2%)	145(2.6%)	0.644	1.128	0.677	1.879
Transport. storage and post	22 (3.6%)	246(4.4%)	0.189	0.724	0.448	1.172
Agriculture	6 (1.0%)	74(1.3%)	0.307	0.638	0.269	1.510
Construction	104 (16.9%)	842(15.1%)	1.000	REF	-	-
Services. including health services	1 (0.2%)	33 (0.6%)	0.221	0.285	0.038	2.131
Other and undetermined	181(29.4%)	1561 (27.9%)	0.745	0.958	0.741	1.239
**Hour of the day**						
7.00–12.00	165 (37.3%)	1907 (45.2%)	1.000	REF	-	-
1.00–6.00	27 (6.1%)	249 (5.9%)	0.527	1.154	0.740	1.799
13.00–18.00	204 (46.2%)	1644 (39.0%)	0.004	1.385	1.112	1.726
19.00–24.00	46 (10.4%)	418 (9.9%)	0.295	1.208	0.848	1.722
Unknown	203 (32.9%)	1815 (32.5%)	-	-	-	-
**Kind of injury**						
Falls to a lower level	33 (5.3%)	322 (5.8%)	0.521	0.877	0.589	1.308
Fall to same level	89 (14.4%)	755 (13.5%)	0.935	1.012	0.763	1.342
Manual handling	90 (14.6%)	871 (15.6%)	0.377	0.883	0.667	1.166
Use of tools/machineries	140 (22.7%)	1190 (21.3%)	1.000	REF	-	-
Others	265 (42.9%)	2448 (43.8%)	0.468	0.923	0.743	1.146
**Prognosis**						
<40 days	545 (88.3%)	4965 (88.9%)	REF	1.000	-	-
≥40 days	72(11.7%)	618 (11.1%)	0.652	1.062	0.818	1.377
Death	0(-)	3 (0.1%)	-	-	-	-

Abbreviations: adjOR (multivariate analysis adjusted odds-ratio); CI (confidence interval); IH (Islamic Holiday); REF (reference).

**Table 5:** Main meteorological indices regarding the assessed time period, broken down by percentiles.

Meteorological Measure (unit)	Min.	Max.	Mean	Percentiles						
				5%	10%	25%	Median	75%	90%	95%
Maximum daily temperature (°C)	0.1	41.2	20.5	5.7	7.5	12.1	21.4	28.6	32.3	33.9
Minimum daily temperature (°C)	−10.5	21.0	6.5	−5.6	−3.6	−0.4	6.8	13.1	16.7	17.9
Average daily temperature (°C)	−6.2	29.9	12.7	−0.6	1.2	4.9	13.1	20.1	24.0	25.3
Relative humidity (%)	14.8	100	65.5	38.4	44.8	55.1	64.6	73.3	83.1	88.4
Solar radiation (kJ/m^2^)	60	245,443	13,867	1,179	2,064	3,373	7,177	12,506	19,854	23,789
Atmospheric pressure (hPa)	911.2	1021.7	985.1	967.6	974.1	981.8	987.1	992.3	998.5	1002.9
Wind Speed (m*s^−1^)	0.1	7.6	1.5	0.6	0.7	0.9	1.4	1.9	2.3	2.6

**Table 6:** Occurrence of Occupational Injuries in EURO and EMRO workers by exposure groups to Air humidity, Atmospheric pressure, Wind Speed, Solar radiation indices broken down by percentiles. Distribution of Occupational Injuries by exposure percentiles and worker groups was assessed through chi squared test (p value; significance level for *p* < 0.05).

	Exposure Percentiles							
	<5%	5–9%	10–24%	25–74%	75–89%	90–94%	>95%	*p* Value
Air Humidity—No. (%)								
EMRO	217 (3.5%)	242 (3.9%)	841 (13.6%)	2667 (43.0%)	1162 (18.7%)	458 (7.4%)	616 (9.9%)	0.067
EURO	5711 (4.2%)	5439 (4.0%)	18766 (13.8%)	58,608 (43.1%)	25,021 (18.4%)	9655 (7.1%)	12,646 (9.3%)	
Atmospheric Pressure—No. (%)								
EMRO	314 (5.1%)	262 (4.2%)	1115 (18.0%)	3008 (48.5%)	842 (13.6%)	342 (5.5%)	319 (5.2%)	0.003
EURO	7751 (5.7%)	6255 (4.6%)	25,429 (18.7%)	65,543 (48.2%)	18,222 (13.4%)	6663 (4.9%)	5983 (4.4%)	
Wind Speed—No. (%)								
EMRO	437 (7.0%)	263 (4.2%)	711 (11.5%)	3333 (53.7%)	866 (14.0%)	296 (4.8%)	297 (4.8%)	0.047
EURO	8567 (6.3%)	5575 (4.1%)	15,094 (11.1%)	72,478 (53.3%)	20,125 (14.8%)	6799 (5.0%)	7207 (5.3%)	
Solar Radiation—No. (%)								
EMRO	398 (6.4%)	359 (5.8%)	793 (12.8%)	1706 (27.5%)	1419 (22.9%)	958 (15.4%)	514 (8.3%)	0.020
EURO	8567 (6.3%)	8159 (6.0%)	16,590 (12.2%)	38,211 (28.1%)	32,364 (23.8%)	19,037 (14.0%)	11,830 (8.7%)	

Abbreviations: EMRO (Eastern Mediterranean); EURO (Europe).

## References

[1] Giraudo M., Bena A., Costa G. (2017). Migrant workers in Italy: An analysis of injury risk taking into account occupational characteristics and job tenure. BMC Public Health.

[2] Kassar H., Marzouk D., Anwar W.A., Lakhoua C., Hemminki K., Khyatti M. (2014). Emigration flows from North Africa to Europe. Eur. J. Publ. Health.

[3] Sweileh W.M. (2018). Global output of research on the health of international migrant workers from 2000 to 2017. Glob. Health.

[4] Schmahl F.W., Metzler B. (1991). The health risks of occupational stress in islamic industrial workers during the Ramadan fasting period. Pol. J. Occup. Med. Environ. Health.

[5] Mertens A., Schouteden M., Godderis L. (2015). Influence of Ramadan on neuroperformance in healthy workers. Med. Res. Arch..

[6] Ovayolu O., Ovayolu N., Tasan E. (2016). Does Ramadan Fasting Affect Fatigue in Nurses?. Holist. Nurs. Pract..

[7] Khalfallah T., Chaari N., Henchi M.A., Abdallah B., Chikh R.B., Saafi M.A., Akrout M. (2004). Evaluation of the impact of Ramadan fasting on the physical workload. Arch. Mal. Prof. Env..

[8] Laraqui S., Manar N., Laraqui O., Caubet A., Verger C., Laraqui C.H. (2012). Influence of Ramadan observance on wakefulness at work among health care workers in Morocco. Arch. Mal. Prof. Env..

[9] Al-Balhan E., Khabbache H., Laaziz A., Watfa A., Mhamdi A., Del Puente G., Bragazzi N.L. (2018). To fast or not to fast during the month of Ramadan? A comprehensive survey on religious beliefs and practices among Moroccan diabetic patients. Diabetes Metab. Syndr. Obes..

[10] Bragazzi N.L. (2014). Ramadan fasting and chronic kidney disease: A systematic review. J. Res. Med. Sci..

[11] Bragazzi N.L., Briki W., Khabbache H., Rammouz I., Mnadla S., Demaj T., Zouhir M. (2015). Ramadan fasting and infectious diseases: A systematic review. J. Infect. Dev. Ctries.

[12] Demirci S., Dogan K.H., Koc S. (2013). Evaluation of forensic deaths during the month of Ramadan in Konya, Turkey, between 2000 and 2009. Am. J. Forensic Med. Pathol..

[13] Aziz A.R., Chia M.Y., Low C.Y., Slater G.J., Png W., Teh K.C. (2012). Conducting an acute intense interval exercise session during the Ramadan fasting month: What is the optimal time of the day?. Chronobiol. Int..

[14] Kjellstrom T., Briggs D., Freyberg C., Lemke B., Otto M., Hyatt O. (2016). Heat, Human Performance, and Occupational Health: A Key Issue for the Assessment of Global Climate Change Impacts. Annu. Rev. Public Health.

[15] Kjellstrom T., Lemke B., Otto M. (2017). Climate conditions, workplace heat and occupational health in South-East Asia in the context of climate change. WHO. South East Asia J. Public Health.

[16] Kjellstrom T., Freyberg C., Lemke B., Otto M., Briggs D. (2018). Estimating population heat exposure and impacts on working people in conjunction with climate change. Int. J. Biometeorol..

[17] Xiang J., Hansen A., Pisaniello D., Bi P. (2016). Workers’ perceptions of climate change related extreme heat exposure in South Australia: A cross-sectional survey. BMC Public Health.

[18] Xiang J., Hansen A., Pisaniello D., Bi P. (2015). Extreme heat and occupational heat illnesses in South Australia, 2001-2010. Occup. Environ. Med..

[19] Xiang J., Bi P., Pisaniello D., Hansen A. (2014). The impact of heatwaves on workers' health and safety in Adelaide, South Australia. Environ. Res..

[20] Otte im Kampe E., Kovats S., Hajat S. (2016). Impact of high ambient temperature on unintentional injuries in high-income countries: A narrative systematic literature review. BMJ. Open..

[21] Riccò M. (2018). Air temperature exposure and agricultural occupational injuries in the Autonomous Province of Trento (2000–2013, North-Eastern Italy). Int. J. Occup. Med. Environ. Health.

[22] Qasrawi S.O., Pandi-Perumal S.R., BaHammam A.S. (2017). The effect of intermittent fasting during Ramadan on sleep, sleepiness, cognitive function, and circadian rhythm. Sleep Breath..

[23] Nugraha B., Ghashang S.K., Hamdan I., Gutenbrunner C. (2017). Effect of Ramadan fasting on fatigue, mood, sleepiness, and health-related quality of life of healthy young men in summer time in Germany: A prospective controlled study. Appetite.

[24] Ajabnoor G.M., Bahijri S., Shaik N.A., Borai A., Alamoudi A.A., Al-Aama J.Y., Chrousos G.P. (2017). Ramadan fasting in Saudi Arabia is associated with altered expression of CLOCK, DUSP and IL-1alpha genes, as well as changes in cardiometabolic risk factors. PLoS ONE.

[25] Laraqui C.H., Tripodi D., Laraqui O., Rahhali A., Caubet A., Daoudi F., Mounassif M., Curtes J.P., Verger C. (2001). The effects of fasting and of the quality of sleep on work during the month of Ramadan. Arch. Mal. Prof. Environ..

[26] Varghese B.M., Hansen A., Nitschke M., Nairn J., Hanson-Easey S., Bi P., Pisaniello D. (2019). Heatwave and work-related injuries and illnesses in Adelaide, Australia: A case-crossover analysis using the Excess Heat Factor (EHF) as a universal heatwave index. Int. Arch. Occup. Environ Health..

[27] Garbarino S., Nobili L., Beelke M., De Carli F., Ferrillo F. (2001). The contributing role of sleepiness in highway vehicle accidents. Sleep.

[28] Binazzi A., Levi M., Bonafede M., Bugani M., Messeri A., Morabito M., Marinaccio A., Baldasseroni A. (2019). Evaluation of the impact of heat stress on the occurrence of occupational injuries: Meta-analysis of observational studies. Am. J. Ind. Med..

[29] Riccò M., Vezzosi L., Gualerzi G. (2018). Health and safety of pesticide applicators in a high income agricultural setting: A knowledge, attitude, practice, and toxicity study from North-Eastern Italy. J. Prev. Med. Hyg..

[30] Rine C.M. (2018). Is Social Work Prepared for Diversity in Hospice and Palliative Care?. Health Soc. Work.

[31] Bahammam A.S., Almushailhi K., Pandi-Perumal S.R., Sharif M.M. (2014). Intermittent fasting during Ramadan: Does it affect sleep?. J. Sleep Res..

[32] Msaad S., Kotti N., Abid S., Hajjaji M., Sellami S., Kammoun S., Yangui J., Masmoudi A. (2016). Influence of Ramadan Observance on Sleep Pattern and Wakefulness at Work among Medical Trainer in Tunisia. J. Sleep Disord. Ther..

